# Identification of *Thalictrum squarrosum* as an alternate host for *Puccinia triticina* and pathogen analysis of *Thalictrum squarrosum* rust

**DOI:** 10.3389/fpls.2025.1566298

**Published:** 2025-04-17

**Authors:** Na Zhao, Liang Huang, Jun Ren, Mengya Zhang, Ting Yi, Hongfu Li, Hao Zhang, Bo Liu, Li Gao, Hongfei Yan, Wanquan Chen, Taiguo Liu

**Affiliations:** ^1^ State Key Laboratory for the Biology of Plant Diseases and Insect Pests, Institute of Plant Protection, Chinese Academy of Agricultural Science, Beijing, China; ^2^ National Agricultural Experimental Station for Plant Protection, Ministry of Agriculture and Rural Affairs, Tianshui, Gansu, China; ^3^ Institute of Millet Crops, Hebei Academy of Agriculture and Forestry Sciences, Shijiazhuang, China; ^4^ College of Plant Protection, Hebei Agricultural University, Baoding, China; ^5^ Control of Biological Hazard Factors (Plant Origin) for Agri-product Quality and Safety, Ministry of Agriculture and Rural Affairs, Beijing, China

**Keywords:** wheat leaf rust, *Puccinia triticina*, *Thalictrum squarrosum*, alternate host, sexual reproduction

## Abstract

*Puccinia triticina* (*Pt*) is a heteroecious fungus needing two different plants as primary and alternate hosts throughout its life cycle. *Thalictrum* spp. were first identified as alternate hosts of *Pt* in 1921, and over 100 species have been identified. However, within China, only *T. petaloideum* L., *T. minus* L., *T. minus* var. *hypoleucum* and *T. baicalense* have been reported as alternate hosts of *Pt*. During the six-year (2015-2018, 2023-2024) field surveys in Zhangbei County (41.26°N, 115.14°E), Zhangjiakou City, Hebei Province, our research team found rust disease on *T. squarrosum*. This persistent infection phenomenon aroused our interest in investigating the role of *T. squarrosum* in the sexual reproduction of *pt*. To clarify whether *T. squarrosum* can serve as an alternate host for *Pt* and to analyze the source of the pathogen, this study used artificial inoculation experiments and molecular identification techniques. The results of the artificial inoculation experiments showed that the basidiospores of *Pt* could infect *T. squarrosum*, and produce pycnia on the adaxial surface of the leaf. Subsequently, aecia were produced on the abaxial side of the leaf after artificial fertilization, and the mature aecia produced aeciospores. The aeciospores were then inoculated into susceptible wheat varieties and the wheat showed typical symptoms of wheat leaf rust. These results confirmed that *T. squarrosum* could serve as an alternate host for *Pt*. For molecular identification, 20 single-aecium samples of *T. squarrosum* were selected. Based on sequence alignment of their ITS regions and phylogenetic analysis, it was shown that rust on *T. squarrosum* could be caused by infection of *Pt* from wheat or the species complex of *P. recondita*. Our study provides new insights into the sexual cycle of *Pt* in China and provides a scientific basis for studying the evolution of *Pt* virulence and optimizing control methods for wheat leaf rust.

## Introduction

Wheat leaf rust, an airborne fungal disease caused by *Pt*, significantly damages wheat leaves. This disease is prevalent in major wheat-producing regions of China, such as the North region, the Huang-Huai-Hai region, the middle-lower reaches of the Yangtze River, the Southwest region, and the Northwest region (Li et al., 2024). Wheat leaf rust not only causes a reduction in the number of grains per spike but also leads to a decline in the quality of wheat grains, resulting in substantial yield losses. In severe cases, the entire harvest may be lost. The most economical and effective method of controlling this disease is through the use of disease-resistant varieties. However, the virulence variation in *Pt* incessantly generates new races, leading to a loss of resistance in several main cultivated wheat varieties in China over a short period. This situation is also significantly increasing the risk of large-scale and devastating outbreaks of wheat leaf rust, thereby posing a serious threat to wheat production and food security.

*Pt* is a representative heteroecious macrocyclic rust fungus. Throughout its life cycle, *Pt* generates five distinct spore types: pycniospores, aeciospores, urediospores, teliospores, and basidiospores. These spores complete complex asexual and sexual reproductive processes on alternate and primary host plants during the life cycle of *Pt* (Li and Zeng, 2002). The asexual phase mainly relies on urediospores to repeatedly infect wheat. In contrast, sexual reproduction is achieved through infections on alternate hosts. This process significantly increases the genetic diversity of the pathogen through the process of meiosis. Furthermore, it enhances the pathogen’s adaptability to host plant defenses and environmental stresses, serving as a key pathway for the emergence of new races (Zhao and Kang, 2023).

Jackson and Mains (1921) performed inoculation experiments that established *Thalictrum* spp. as alternate hosts for *Pt*, thus initiating the investigation into alternate hosts for this pathogen. *Thalictrum* spp. comprises about 220 species, mainly in temperate and cool temperate zones of Asia, Europe, Africa, North America, and South America, with a small number of species located in subtropical regions (Wang, 2018). According to recent reports, 17 species of *Thalictrum* have been identified as alternate hosts for *Pt* (Zhao et al., 2021; Anikster et al., 1993; Davis, 1963; Mains, 1932). In addition, several species within the genera *Isopyrum*, *Anchusa*, *Clematis*, and *Echium* have been recognized as alternate hosts for *Pt*. However, these alternate host species exhibit regional variations (Chester, 1946; Sibilia, 1960; D’Oliveira and Samborski 1966).

Since the early 1990s, researchers in China have started to study the alternate hosts of *Pt*, but there have been relatively few studies to date. By means of artificial inoculation, researchers have only confirmed that *T. petaloideum* L. and *T. minus* L. in Inner Mongolia Autonomous Region, *T. minus* var. *hypoleucum* in Shaanxi, and *T. baicalense* in Gansu can serve as the alternate hosts for *Pt* (Zhao et al., 1994, 2021). During the six - year period (2015-2018, 2023-2024), our research team investigated the rust disease on *Thalictrum* plants in Zhangbei County, Zhangjiakou City, Hebei Province. An unknown species of *Thalictrum* was found to be infected with rust, and the disease occurrence was persistent and stable. This finding suggests that this *Thalictrum* species may have the potential to be an alternate host for *Pt*. This experiment aims to identify the *Thalictrum* species, determine whether it acts as an alternate host for *Pt*, and analyze the rust pathogen by combining morphological identification, artificial inoculation experiments, and molecular identification, thereby providing a new scientific basis for the prevention and control strategy of wheat leaf rust.

## Materials and methods

### Disease survey and sample collection

During field surveys for rust disease of *Thalictrum* species in Zhangbei County (41.26°N, 115.14°E), Zhangjiakou City, Hebei Province, an unknown *Thalictrum* species with rust infection was discovered. To fully assess the prevalence and severity of rust disease, the diseased leaf incidence, diseased plant incidence and disease severity were calculated using the method of random sampling. For each sampling, 30 leaves and 20 *Thalictrum* plants were selected. The sampling was repeated 5-8 times. Diseased leaf incidence was obtained by counting the number of leaves with rust symptoms and dividing by the total number of leaves examined in a randomly selected sample of *Thalictrum* plants. Diseased plant incidence was determined by counting the number of infected *Thalictrum* plants and dividing by the total number of plants surveyed at each location. Disease severity was assessed by the percentage of leaf area covered by rust lesions on diseased *Thalictrum* leaves.

From these infected *Thalictrum* plants, branches with mature and fresh aecia were collected using sterilized tools. They were placed in self-sealing bags and stored at 4°C. Concurrently, seeds were collected and seedlings were transplanted for subsequent pathogenicity testing. Typical photographs of *Thalictrum* plants were taken and species identification was based on morphological characteristics such as floral structures; leaf morphology; and achene types. The identification process followed the standard methodology of *Flora of China* (www.iplant.cn/), supplemented by *Thalictrum* (*Ranunculaceae*) *in China* (Wang, 2018).

### Teliospore germination

Teliospore germination tests were performed in accordance with the method described by Kang (2016), using wheat leaves stored in the laboratory. These leaves contained teliospores produced by artificial inoculation with *Pt*. Wheat leaves were cut into leaf segments (5 cm) and rinsed three times with sterile distilled water. These leaf segments were then placed in sterile Petri dishes (90 mm diameter) with double-layered filter paper. Two cycles of alternating wet and dry incubation were performed in an incubator under the following conditions: a 12 hours dark phase at 10°C with 100% relative humidity, followed by a 12 hours light phase at 16°C with 5000 lux illumination. After 48 hours, leaf segments were chopped and evenly distributed on water agar medium (1.5-2% w/v, pH 6.8) for continued alternating dry-wet cycles cultivation. Each germination test was repeated three times. Regular observations were made under the light microscope and inoculation was performed when teliospores were observed to germinate and produce basidiospores.

### Inoculation of *Thalictrum* plants with basidiospores

*T. squarrosum* seeds were surface sterilized (0.1% NaClO, 3 min), and hydroclimatized in sterile Petri dishes (60 mm diameter) with sterile distilled water then incubated in an incubator (20°C, 100% RH, 16/8 h photoperiod). After about 20 days, the seeds germinated and cotyledons developed, they were transplanted into small pots (20 cm diameter) and grown in a sterile greenhouse (20°C, 60% RH, 16/8 h photoperiod). About 30 days after transplantation, the seedlings produced about 5-10 true leaves. The newly emerged leaves were tender and more susceptible to infection by basidiospores.

For inoculation, *T. squarrosum* leaves and teliospore-germinated Petri dishes were sprayed with sterile distilled water. The Petri dishes were then placed directly above the *T. squarrosum* plants to make the basidiospores to fall onto and infect the *T. squarrosum* leaves. The inoculated *T. squarrosum* plants and teliospore-germinated Petri dishes were co-incubated in a dark inoculation room (16°C, 100% RH) for 72 hours and then transferred in a sterile greenhouse (16°C, 60% RH, 16/8 h photocycle). Each inoculation experiment was repeated three times. The inoculated *T. squarrosum* leaves were regularly observed for infection symptoms and the mature aecia were used as inoculum for wheat seedlings.

### Inoculation of wheat plants with aeciospores

The susceptible wheat cultivar Zhengzhou 5389 was sown in a plastic tray (6 cm×7cm×8cm) and was inoculated at the one-leaf-one-heart stage. The aecium collected from *T. squarrosum* leaves infected with basidiospores was placed on glass slides and gently pressed with inoculation needles to release aeciospores. Sterile distilled water was then added to create a spore suspension (1×10^4^ aeciospores/mL). This suspension was then inoculated onto dewaxed wheat leaves using inoculation needles and cultured in a dark inoculation room (20°C, 100% RH) for 24 hours, and then transferred to a greenhouse (20°C, 100% RH, 16/8 h photoperiod) for further cultivation. From 16 to 20 days after inoculation, the wheat leaves were continuously observed for typical symptoms of wheat leaf rust. All wheat inoculation experiments were repeated three times.

### Molecular identification and phylogenetic analysis

Genomic DNA was extracted from aeciospores using an adapted CTAB method as described by Gill et al. (1991). PCR amplification was performed using universal fungal primers ITS3 (GCATCGATGAAGAACGCAGC) and IT4 (TCCTCCGCTTATTGATATGC). The PCR products were then subjected to electrophoresis on a 1.0% (w/v) agarose gel in 1×TAE buffer. After separation, the DNA bands were visualized using a UV transilluminator. Amplification bands were excised from the gel and integrated into the T-Vector pMD™19 carrier (Takara Biotechnology (Dalian) Co., Ltd., Japan). Sequencing was performed by Beijing Qingke Biological Technology Co., Ltd. (Beijing, China). The final sequences were aligned with the NCBI database (https://www.ncbi.nlm.nih.gov/) for species identification.

The phylogenetic and molecular evolutionary analyses were conducted using the neighbor-joining (NJ) method found in the Molecular Evolutionary Genetics Analysis (MEGA 11) package (Tamura et al., 2021). Data were analyzed using Poisson correction, and gaps were removed by complete deletion. The topological stability of the neighbor-joining tree was evaluated by 1000 bootstrap replications. The final phylogenetic tree was modified and beautified on the tvBOT: Tree Visualization by One Table (https://www.chiplot.online/tvbot.html).

## Results

### Identification of *Thalictrum* specie

Based on the morphological characteristics of the flowers, leaves, and achenes, the unknown *Thalictrum* species collected in Zhangbei County, Zhangjiakou, Hebei Province, was identified as *T. squarrosum*. The flowers were yellowish-green in color and long elliptical or nearly circular in shape. The inflorescences are umbrella-cone-shaped with nearly dichotomous branches. The stem is between 60-100cm in height, with bipinnate to tripinnate pinnately compound leaves, and leaf blades between 8-18cm in length. The achenes are fusiform in shape.

**Figure 1:**
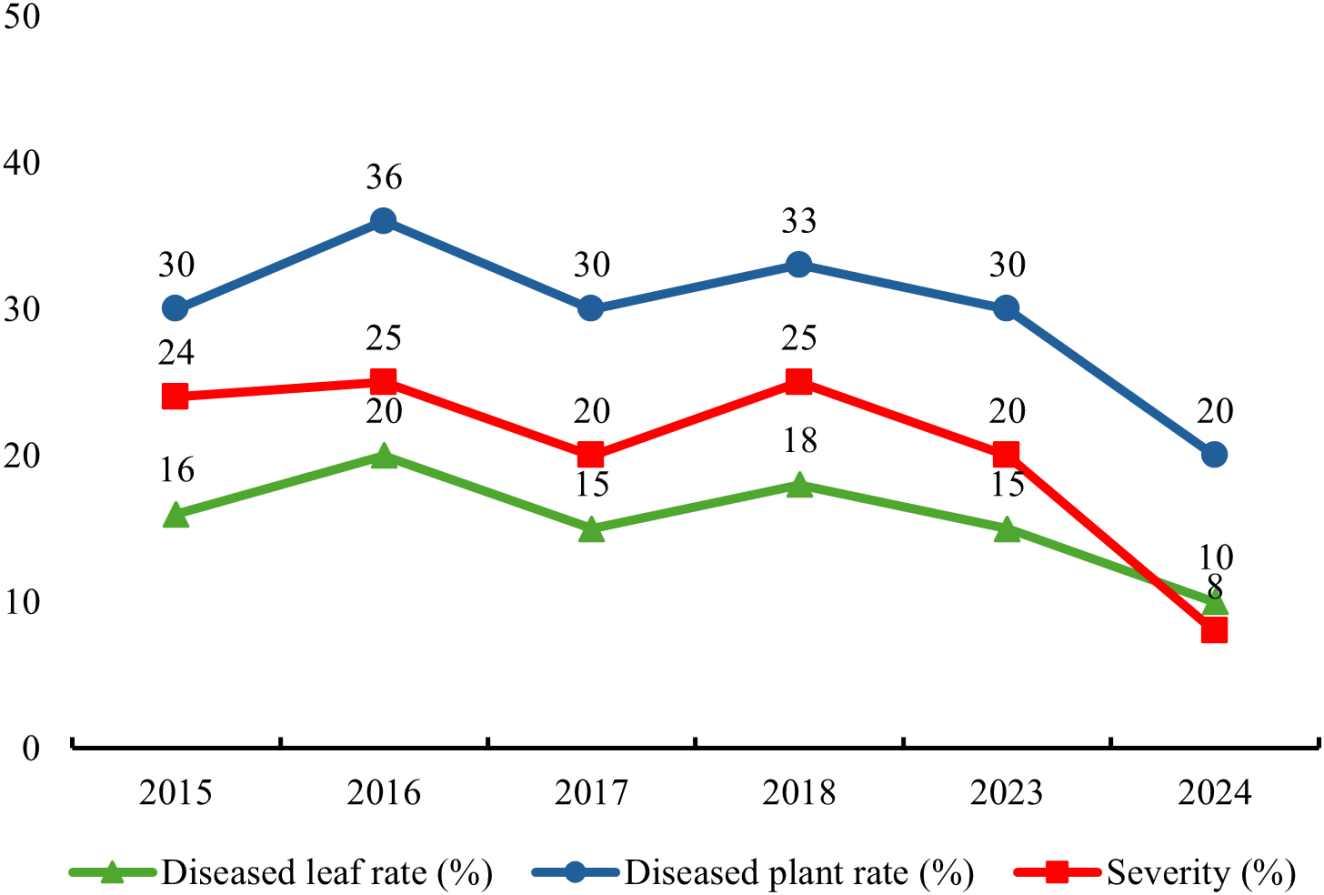
Statistical graph of the diseased leaf incidence, diseased plant incidence and severity of rust disease on *T. squarrosum.* Diseased leaf incidence represents the proportion of leaves with rust symptoms among the total examined leaves of *T. squarrosum* plants. It fluctuated 15%-20% from 2015-2023 and dropped to 8% in 2024. Diseased plant incidence is the proportion of infected *T. squarrosum* plants among the total surveyed plants. It ranged 30%-36% in 2015-2023 and decreased to 20% in 2024. Disease severity is measured by the percentage of leaf area covered by rust lesions on diseased leaves. It was 20%-25% from 2015-2023 and dropped to 10% in 2024.

**Figure 2:**
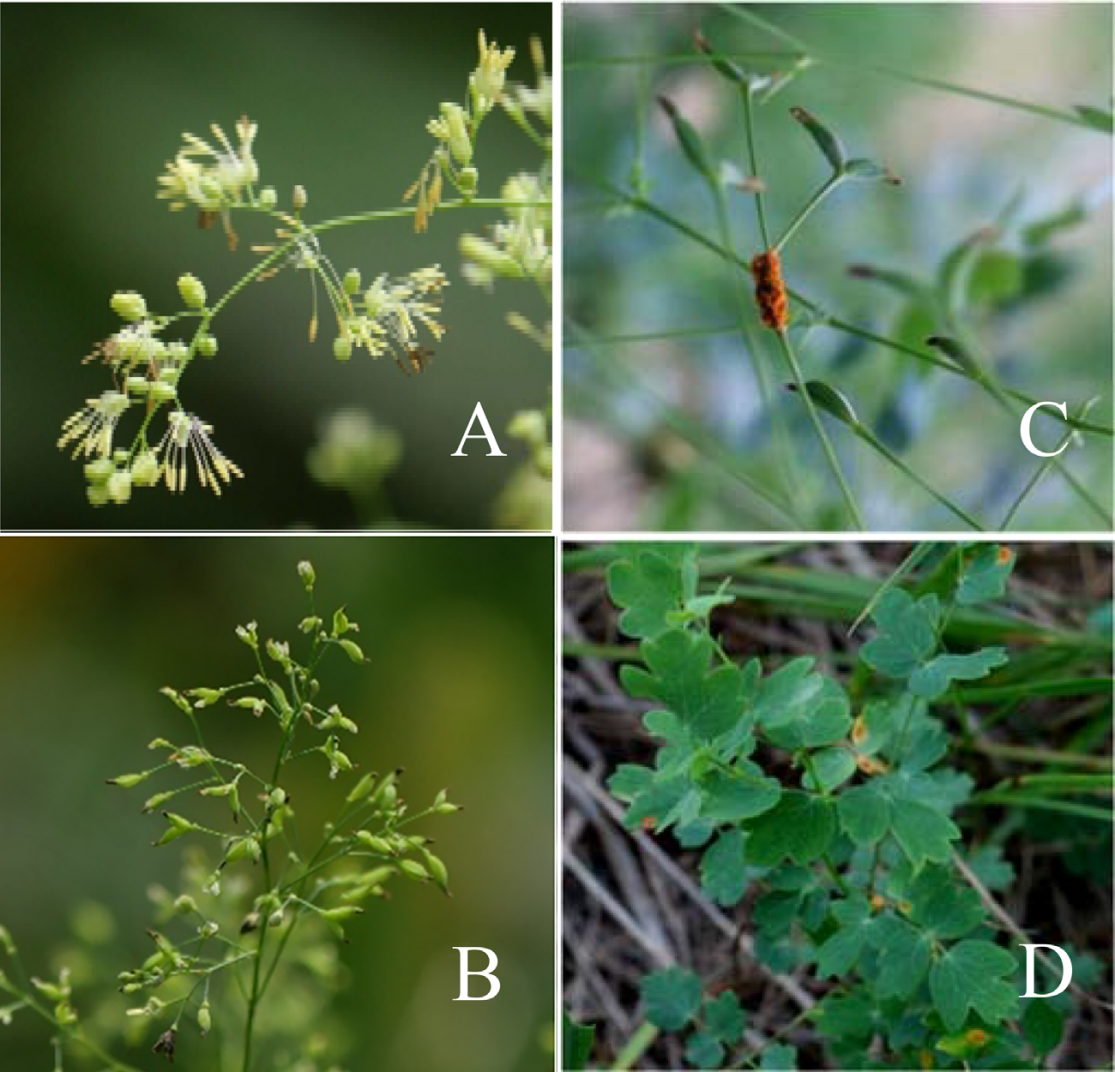
Morphology and rust symptoms of *T. squarrosum*. **(A)** The flowers of *T. squarrosum*; **(B)** The achenes; **(C, D)** The symptoms of rust disease on the stems and leaves of *T. squarrosum*.

A systematic survey and statistical work on the diseased leaf incidence, diseased plant incidence and severity of *T. squarrosum* rust were also conducted, and the detailed data are presented in **Figure 1**. From 2015 to 2023, the diseased leaf incidence was between 15% and 20%, the diseased plant incidence was between 30% and 36%, and the severity was between 20% and 25%. By 2024, all three measures had decreased substantially, with the diseased leaf incidence, diseased plant incidence, and disease severity dropping to 10%, 20%, and 8%, respectively. This is closely related to the environmental factors of the year, as there was little rainfall and a dry climate in the area in 2024, making it less conducive to the occurrence and development of the disease. In general, the indicators fluctuated within a certain range, indicating that the incidence of *T. squarrosum* rust was relatively stable in the region. The main site of infection is concentrated on the leaves, stems, and achenes of *T. squarrosum* plants, and the spots showed various shapes. **Figure 2** clearly shows the morphology of *T. squarrosum* and the symptoms of natural rust disease.

### The identification of alternate host for *Pt*

**Figure 3:**
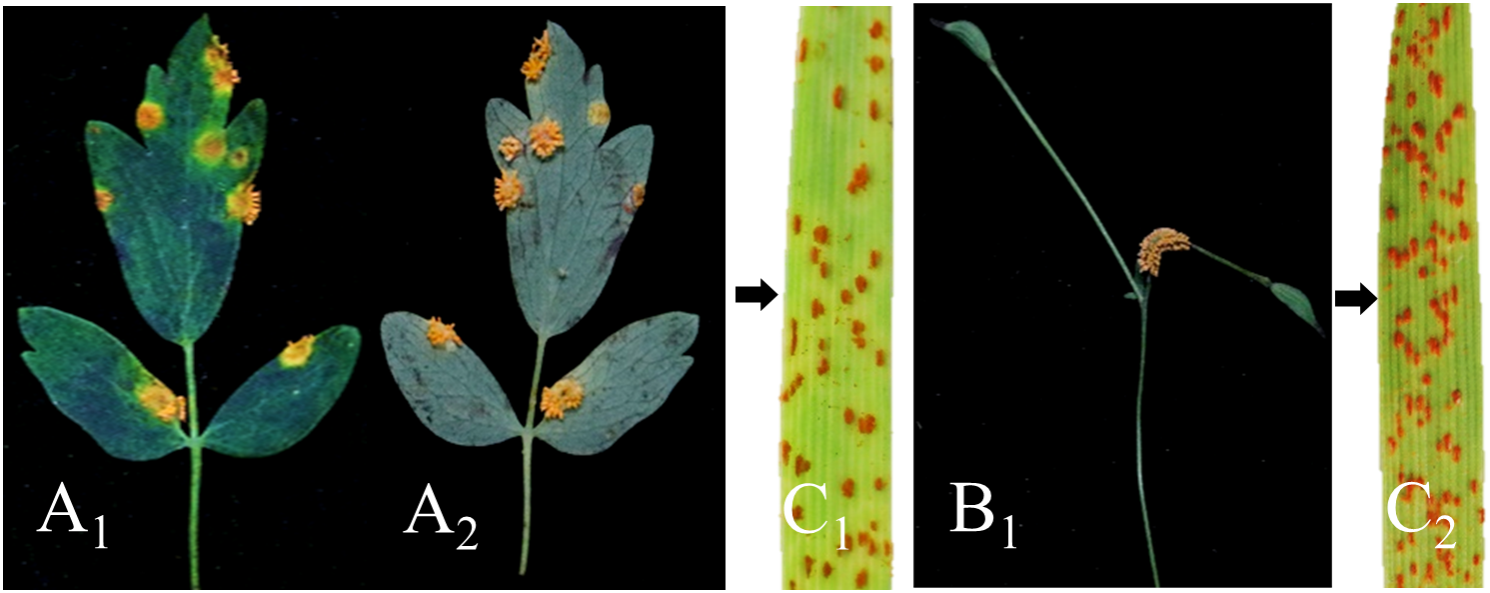
Confirmation of *T. squarrosum* as an alternate host for *Pt* through artificial inoculation. **(A_1_)** Pycnial stage; **(A_2_)** Aecia formed on the lower surface of leaves; **(B_1_)** The disease developed in the stems; **(C_1_, C_2_)** The typical symptoms of wheat leaf rust appeared after inoculation of wheat with aeciospores.

The basidiospores derived from the germinated teliospores of *Pt* were used to inoculate *T. squarrosum*. At 13-15 days after inoculation, we observed the formation of pycnia on the adaxial surface of the leaf. These pycnia appeared as a yellow, gelatinous, viscous liquid containing pycniospores. At 21-23 days, tubular aecia developed at the corresponding infection site on the abaxial surface (**Figure 3**). Mature aeciospores were then harvested from the aecium and used to inoculate wheat. These wheat plants developed typical symptoms of wheat leaf rust within 12-14 days. The infected wheat leaves produced randomly distributed yellowish-brown urediospores (**Figure 3**). The results indicated that *Pt* was able to infect *T. squarrosum* and complete the entire sexual phase of its life cycle, suggesting that *T. squarrosum* could serve as an alternate host for *Pt*.

### Molecular identification of aecium samples on *T. squarrosum*

To determine whether the aeciospores on *T. squarrosum* were resulted from *Pt* infection under natural conditions, we collected 20 single-aecium samples from *T. squarrosum*, and amplified a single 401 bp band in each genomic DNA using the universal fungal primers ITS3 and ITS4. Alignment of the ITS sequences from these 20 samples (**Table 1**) with the NCBI database revealed over 96% homology with *Pt* (GenBank accession numbers DQ460724, DQ417410, DQ417419 and AF511083). The results indicated that under natural conditions, rust on *T. squarrosum* could be caused by *Pt* infection.

**Table 1:** Sequence alignments of 20 aecial samples collected from *T. squarrosum* based on the internal transcribed spacer (ITS) region, showing high identities with *Puccinia triticina* sequences in the NCBI database.

No.	Sample code	Size of sequence/bp	Identity with submitted sequence at GenBanka ^a^/%	GenBank accession	No.	Sample code	Size of sequence/bp	Identity with submitted sequence at GenBanka ^a^/%	GenBank accession
1	TP2401	401	96	PQ524479	11	TP2411	401	96	-^b^
2	TP2402	401	96	-^b^	12	TP2412	401	96	-^b^
3	TP2403	401	96	-^b^	13	TP2413	401	96	PQ524482
4	TP2404	401	96	-^b^	14	TP2414	401	96	-^b^
5	TP2405	401	96	PQ524480	15	TP2415	401	96	-^b^
6	TP2406	401	96	-^b^	16	TP2416	401	96	PQ524483
7	TP2407	401	96	-^b^	17	TP2417	401	96	-^b^
8	TP2408	401	96	-^b^	18	TP2418	401	96	-^b^
9	TP2409	401	96	-^b^	19	TP2419	401	96	-^b^
10	TP2410	401	96	PQ524481	20	TP2420	401	96	PQ524484

a Sequences of 20 aeciospores samples from *T. squarrosum* shared highest similar identity with that of *P. triticina* isolate (accession number AF511083), which was not listed in this table.

b ITS sequences of 20 aeciospores from *T. squarrosum* were almost identical with the sequences of samples of TP2401, TP2405, TP2410, TP2413, TP2416, TP2420 in the GenBank of NCBI database.

### Phylogenetic analysis

**Figure 4:**
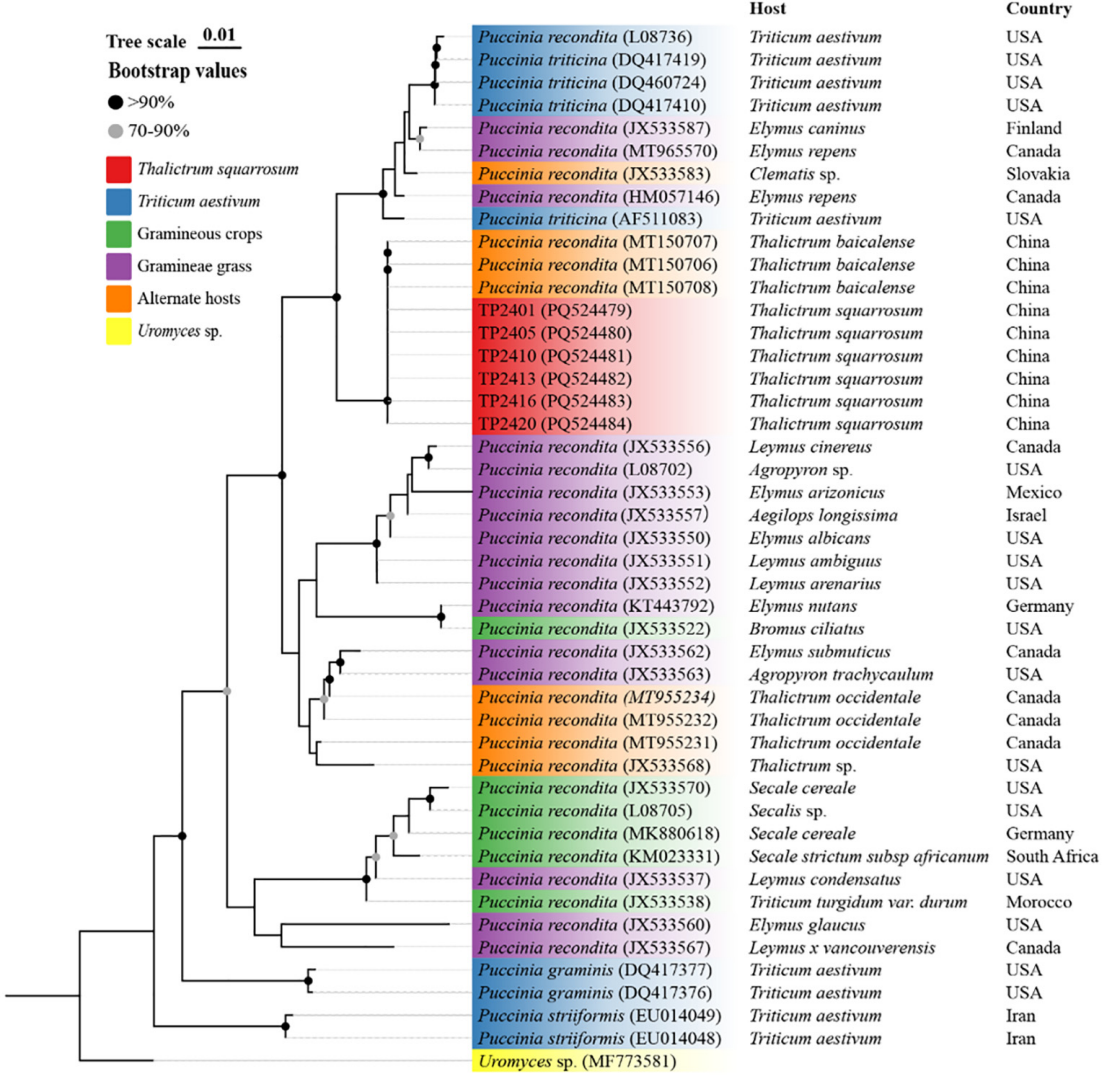
Phylogenetic analysis of sequences from six selected single-aecium samples of *T. squarrosum* (PQ524479-PQ524484) submitted to the GenBank of NCBI database.

Six single-aecium samples from naturally infected *T. squarrosum* plants, along with those of fungus on wheat, grasses, alternate host plants, related *Puccinia* species, and *Uromyces* sp., were sourced from the NCBI database to construct a phylogenetic tree (**Figure 4**). The phylogenetic analysis revealed that the sequences of the six single-aecium samples (PQ524479-PQ524484) on *T. squarrosum* closely aligned with those of *P. recondita* on *T. baicalense* (MT150706-MT150711), indicating a high degree of genetic similarity. Furthermore, the selected single-aecium samples of *T. squarrosum* and *P. recondita* on *Elymus* spp. (JX533587, MT965570, HM057146), wheat (L08736, DQ417419, DQ460724, DQ417410, AF511083), and *Clematis* sp. (JX533583) were classified within the same subclade. Significantly, *P. triticina* from wheat (DQ460724, DQ417410, DQ417419 and AF511083) exhibited greater sequence homology with single-aecium samples from *T. squarrosum*. The results indicated that *T. squarrosum* rust could be caused by the infection of *Pt* from wheat or the species complex of *P. recondita*.

## Discussion

Alternate hosts play a critical role in the sexual reproduction of *pt*. The germination degree of teliospores is one of the key factors in determining whether alternate hosts can be effective. In 1986, Zhao et al. (1994) found severe rust disease on *T. petaloideum* L. and *T. minus* L. in Zhuozhi County, Inner Mongolia, and began research on the alternate hosts of *Pt* in China. Through artificial inoculation, they demonstrated that *T. petaloideum* and *T. minus* L. could serve as alternate hosts of *Pt*. However, *T. minus* var. *stipellatum* and *T. minus* var. *hypoleucum* did not produce rust disease symptoms after inoculation. However, Zhao et al. (2021) confirmed that *T. minus* var. *hypoleucum* and *T. baicalense* were also alternate hosts. At the beginning of the study, due to the lack of a mature and efficient teliospore germination technique, researchers tried various methods such as freeze-thaw alternation, dry-wet alternation and gibberellin treatment to promote teliospore germination (Wang et al., 1987). The experimental results showed that only the dry-wet alternation method could induce germination of individual teliospores. This low germination rate was probably responsible for the failure of *T. minus* var. *hypoleucum* to develop disease symptoms during the early inoculation trials. In this study, the probability of teliospore germination was increased to 35-45% using the optimized dry-wet alternation method proposed by Kang (2016), and basidiospores were successfully inoculated onto *T. squarrosum*. The aeciospores produced on *T. squarrosum* were then inoculated onto wheat and showed typical symptoms of wheat leaf rust. This finding confirms that *T. squarrosum* can serve as an alternate host for *pt* in China. In addition, the optimized dry-wet alternation method provides valuable technical support for the identification of alternate hosts.

*Thalictrum* plants may serve as alternate hosts for *Pt* or other rust fungi. Our laboratory has successfully inoculated susceptible wheat varieties with aeciospores collected in the field from *T. squarrosum* aecia and produced typical symptoms of wheat leaf rust, confirming that *Pt* is one of the pathogenic fungi responsible for *T. squarrosum* rust in the natural environment (Unpublished). Kang (2016), utilizing the specific molecular marker EST-6, sought to detect *Pt* on *Thalictrum* plants in various locations including Zhuozhi County in Inner Mongolia, and Chengde and Chongli Counties in Hebei Province. The detection rates of *Pt* on *Thalictrum* plants varied, with 55% in Chengde, 45% in Chongli, and a mere 4% in Zhuozhi. Molecular evidence pointed towards *Thalictrum* spp. as the alternate host of *Pt*, though regional differences were apparent and the possibility of other rust fungi existing on *Thalictrum* plants was noted. The aforementioned research also suggests that *Pt* may, under natural conditions, infect susceptible *Thalictrum* plants to complete its sexual cycle in China. Huang et al. (1956) discovered that aeciospores from *T. simplex* were unable to infect wheat in artificial inoculation experiments conducted in Jiamusi City, Heilongjiang Province. However, they were able to infect *Elymus sibiricus*, and the rust observed on *T. simplex* could be attributed to *P. rubigo-vera*. Wang et al. (1987) reported that numerous species of weeds coexist with *Thalictrum* in the same environment. This suggests an association between the occurrence of rust on *Thalictrum* and its presence on grass weeds. Furthermore, *T. minus* L. and *T. petaloideum* L. were identified as alternate hosts for *Agrostis clavata* Trin. and *Bromus inermis* Leyss. Zhao et al. (2021) conducted phylogenetic analyses on the ITS region sequences of six single-aecium samples of *T. baicalense*, comparing them with *Pt* on wheat and P*. recondita* on various hosts. The findings revealed that *P. recondita* on *T. baicalense*, *Pt* on wheat, *P. recondita* on *Elymus repens*, *P. persistens* on *T. minus* L., and *P. recondita* on *Clematis* sp. were all part of a closely related subgroup. *P. recondita* is a collective term for leaf rust fungi exhibiting similar spore morphology on wheat and other distant hosts, including several grass species, wild wheat, and rye (Cummins and Caldwell, 1956). In this research, the sequences of single-aecium samples from *T. squarrosum* exhibited 99-100% similarity to sequences from *T. baicalense* aecium (MT150706-MT15079). Moreover, evolutionary tree analyses largely aligned with these findings. These data suggest that *Thalictrum* plants are susceptible to rust infection from a range of rust fungi, including those from various wheat species, grass crops, and weeds.

Alternate hosts have been deemed capable of functioning in some regions, with varying effects on the incidence and epidemiology of wheat leaf rust (Wang, 1985). For instance, in eastern Siberia, the urediospores of *Pt* were unable to overwinter due to environmental constraints. Consequently, aeciospores produced by *Isopyrum fumaroides* became the sole source of wheat leaf rust infection within this region, although they do not operate outside of it (Chester, 1946). In North America, *Pt* can successfully infect *Thalictrum* spp. and *Isopyrum* spp. plants under artificial conditions, but these infections do not occur naturally (Levine and Hildreth, 1957; Sarii et al., 1968). In China, Huang et al. (1956) and Wang et al. (1987) conducted field experiments which concluded that *T. simplex*, *T. minus* L. and *T. petaloideum* L. did not serve as effective alternate hosts for *Pt* under natural conditions. And recent research by Zhao et al. (2021) has shown that under natural conditions, *Pt* in China may complete its sexual cycle by infecting susceptible *Thalictrum* plants. However, direct evidence to confirm this conclusion is still lacking, necessitating further research.

In China, the *Thalictrum* species boasts a rich diversity, encompassing approximately 100 species and 20 varieties spread across 29 provinces (Wang, 2018). However, the technical challenges of artificially germinating teliospores and the susceptibility of the infection process to environmental factors have limited studies on the alternate hosts of *Thalictrum* species for *Pt*. Combined with the results of this study, five *Thalictrum* species were identified as alternate hosts for *Pt* in China: *T. petaloideum* L., *T. minus* L., *T. minus* var. *hypoleucum*, *T. baicalense* and *T. squarrosum*. The existence of other potential alternate hosts for *Pt* remains uncertain and warrants further research. These investigations will not only shed light on the transmission pathway and genetic variation mechanism of *Pt*, but also provide a scientific foundation for developing effective disease prevention and control strategies to ensure the production safety of wheat crops.

## Data Availability

The datasets presented in this study can be found in online repositories. The names of the repository/repositories and accession number(s) can be found in the article/supplementary material.
